# A study of splicing mutations in disorders of sex development

**DOI:** 10.1038/s41598-017-16296-3

**Published:** 2017-11-24

**Authors:** Flavia Leme de Calais, Lindsay D. Smith, Michela Raponi, Andréa Trevas Maciel-Guerra, Gil Guerra-Junior, Maricilda Palandi de Mello, Diana Baralle

**Affiliations:** 1 Human Development and Health, Faculty of Medicine, University of Southampton, Southampton, UK; 2 Centro de Biologia Molecular e Engenharia Genética, Universidade Estadual de Campinas, Campinas, Brazil; 3 Cancer Sciences Unit, Faculty of Medicine, University of Southampton, Southampton, UK; 4 Departamento de Genética, Faculdade de Ciências Médicas, Universidade Estadual de Campinas, Campinas, Brazil; 5 Departamento de Pediatria, Faculdade de Ciências Médicas, Universidade Estadual de Campinas, Campinas, Brazil; 6 Wessex Clinical Genetics Service, Southampton University Hospitals NHS Trust, Southampton, UK

## Abstract

The presence of splicing sequence variants in genes responsible for sex development in humans may compromise correct biosynthesis of proteins involved in the normal development of gonads and external genitalia. In a cohort of Brazilian patients, we identified mutations in *HSD17B3* and *SRD5A2* which are both required for human sexual differentiation. A number of these mutations occurred within regions potentially critical for splicing regulation. Minigenes were used to validate the functional effect of mutations in both genes. We evaluated the c.277 + 2 T > G mutation in *HSD17B3*, and the c.544 G > A, c.548-44 T > G and c.278delG mutations in *SRD5A2*. We demonstrated that these mutations altered the splicing pattern of these genes. In a genomic era these results illustrate, and remind us, that sequence variants within exon-intron boundaries, which are primarily identified for diagnostic purposes and have unknown pathogenicity, need to be assessed with regards to their impact not only on protein expression, but also on mRNA splicing.

## Introduction

Discovered in the late 1970s, the splicing process is a crucial step in the biogenesis of eukaryotic mRNA^1,2^. The chemistry of the splicing reaction is mediated by the spliceosome, a ribonucleoprotein complex assembled from five subcomplexes called snRNP’s (U1snRNP, U2, U4, U5 and U6)^3^ which interact with specific sequences at the exon/intron boundaries to direct intron excision and exon ligation to produce the mature mRNA^4^.

The first core splicing signal sequence is the 5′ splice site, which is present at the 5′ end of the intron and includes a universally conserved GU dinucleotide (the donor site AG/GURAGU, where R is a purine). The second consensus sequence is the 3′ splice site, located at the 3′ end of the intron and defined by three conserved elements: the branch point (composed of the sequence YNYURAC, where Y is a pyrimidine), polypyrimidine tract, and terminal AG at the extreme 3′ end of the intron (the acceptor site YAG/RNNN)^5,6^.

Auxiliary cis-elements, known as exonic and intronic splicing enhancers (ESEs and ISEs) and silencers (ESSs and ISSs), are essential sequences which increase splicing fidelity^7^. Many ESEs and ISEs contain binding sites for members of the SR protein family. SR proteins are often involved in positive regulation of the splicing process and promote early spliceosomal complex assembly^8^. Whereas, the best characterized splicing silencers are recognized by heterogeneous nuclear ribonucleoproteins (hnRNPs)^9^.

Cis-acting mutations can disrupt the use of essential splice sites resulting in loss of gene expression or activation of alternative splice sites, which force the expression of alternative mRNA isoforms. Importantly, if the expression of alternative splicing isoforms occurs at an inappropriate developmental stage this may result in disease^10^. Alterations in the spliceosome machinery and point mutations located at intron-exon boundaries can result in severe changes to the splicing processe and thus impact on the appearance of disease^11^. It has been shown that 15–62% of all splicing mutations result in the expression of aberrant mRNA products that directly result in disease^12–14^.

In the present study we have evaluated the effect of mutations within two genes critical for human sexual development: *HSD17B3* and *SRD5A2*. These genes are involved in the biosynthesis of steroid hormones, testosterone (T) and dihydrotestosterone (DHT) respectively. The *HSD17B3* gene produces the 17beta-hydroxysteroid dehydrogenase type 3 (17beta-HSD3) enzyme, which catalyzes the synthesis of T from androstenedione (A). The *SRD5A2* gene encodes the 5alpha-reductase type 2 enzyme, which converts T to DHT, a more active metabolite^15,16^. Individuals with 17beta-HSD3 or 5alpha-reductase type 2 deficiencies will experience undermasculinization with predominantly female external genitalia or may present hypospadias. They are often raised as girls despite the fact that they carry a 46,XY karyotype. Deficiencies in either 17beta-HSD3 or 5alpha-reductase type 2 enzymes are inherited as autosomal recessive disorders^17,18^. A limited number of mutations within 5′ and 3′ splice sites have been described for these genes^19–22^.

The aim of this study was to functionally evaluate the effect of sequence variants of unknown significance (VUS) within these genes which were predicited to affect splicing: c.277 + 2 T > C in *HSD17B3*, and c.278delG, c.183-44 T > G and c.544 G > A in *SRD5A2*. For this purpose we developed hybrid minigenes containing either wild-type (WT) DNA sequences or DNA from patients carrying the sequence variants. Assessment of the splicing pattern of either WT or mutant minigenes enabled us to determine the effect of the VUS on the splicing mechanism.

## Results

To functionally validate whether the identified sequence variants disrupted splicing, minigenes containing the mutation of interest or the WT sequence were constructed. Each minigene construct was transfected, in triplicate, into HEK-293 cells. 48 hours later cells were harvested, RNA purified and the effect on splicing determined by semi-quantitative RT-PCR and sequencing.

**Figure 1:**
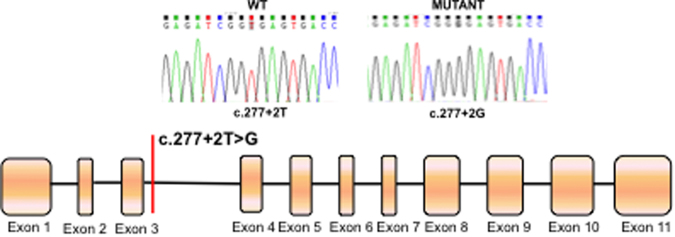
Schematic diagram showing the location of the c.277 + 4 A > T mutation in the *HSD17B3* gene. Boxes represent exons, lines represent introns.

**Figure 2:**
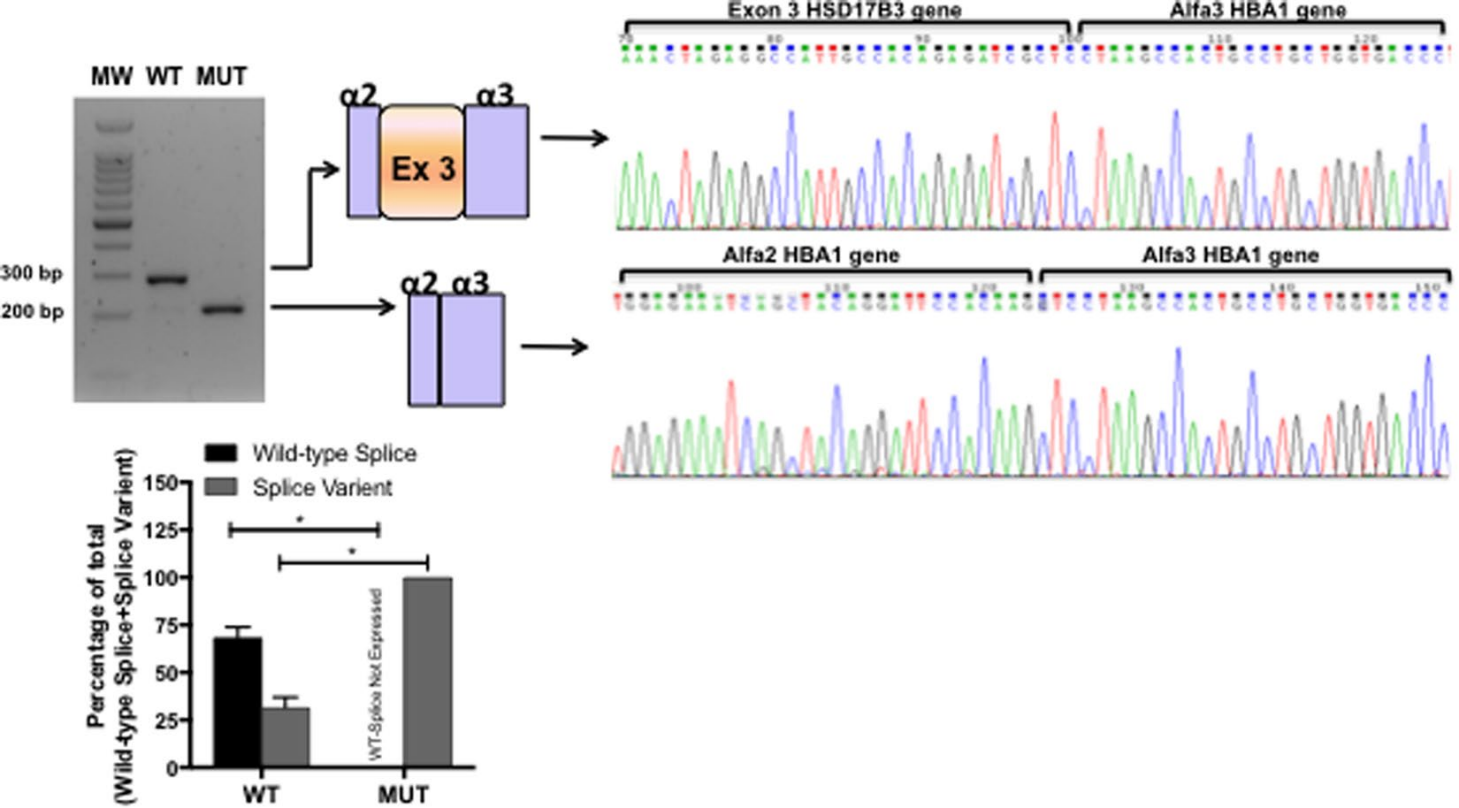
RT-PCR results from transfections of wild-type *HSD17B3* or c.277 + 2 T > G *HSD17B3* minigenes. A representative agarose gel (n = 3) showing the results of RT-PCR of wild-type (WT) and mutant (MUT) minigene splicing is shown and quantified for the accompanying graph. Schematic diagrams identifying wild-type splicing (293 bp) and exon 3 skipping (217 bp) are shown. The panel on the right shows electropherograms of cDNA splicing isoforms generated from wild-type and mutant minigenes. The histogram shows the relative percentage expression of each isoform calculated against total intensity of the bands representing each isoform. Student t test analysis shown; *p < 0.01.

The c.277 + 2 T > G *HSD17B3* mutation located in the 5′ splice site of intron 3 (Fig. 1) was previously described in compound heterozygosis with the c.277 + 4 A > T mutation in a 46,XY female patient^23^. As aberrant splicing had already been described for the c.277 + 4 A > T *HSD17B3* mutation^19^, we investigated the affect of the c.277 + 2 T > G mutation on splicing. RT-PCR results showed that the c.277 + 2 T > G mutation enhanced skipping of exon 3 compared to WT (Fig. 2).

**Figure 3:**
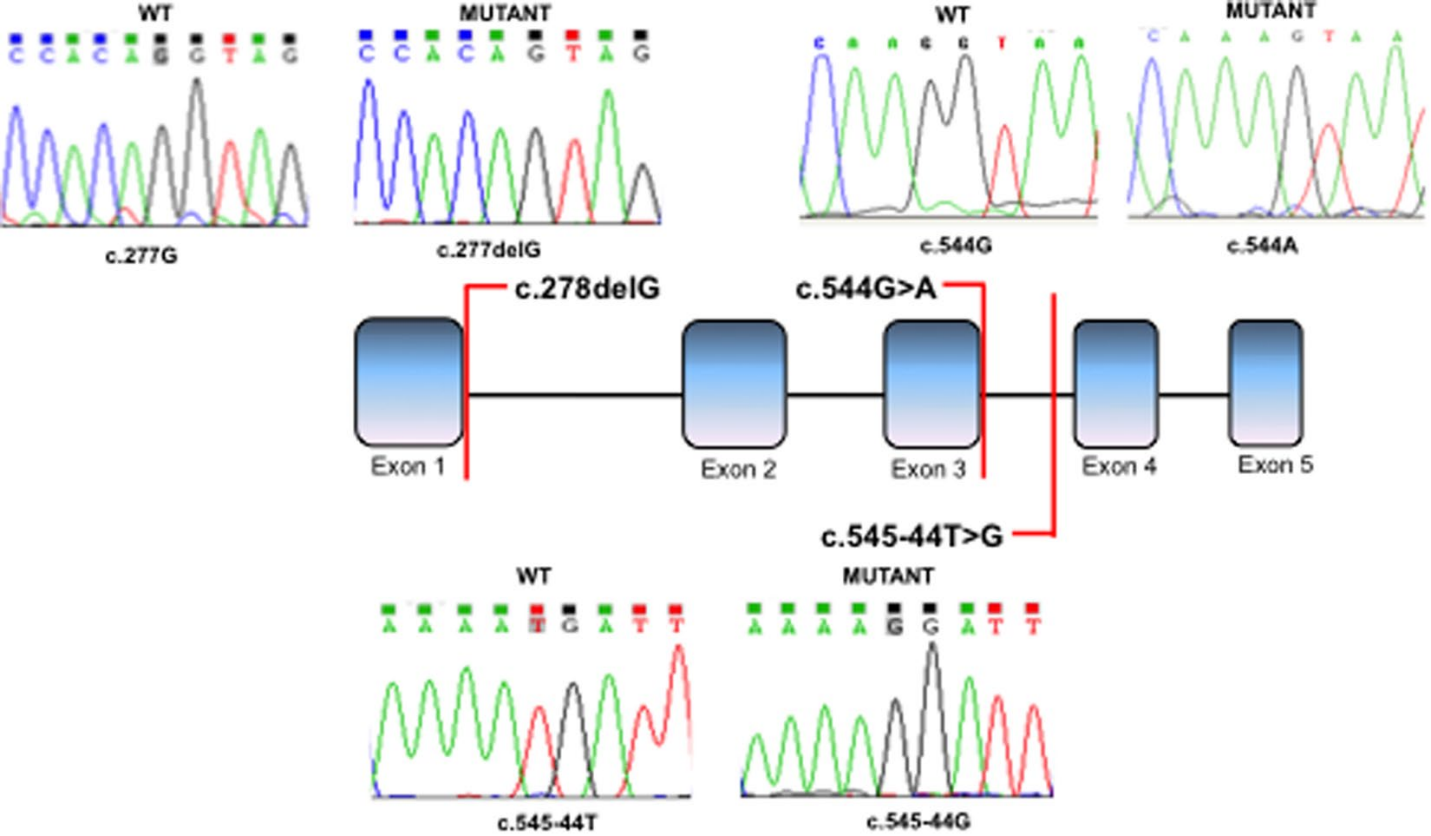
Schematic diagram showing the location of c.278delG, c.183-44 T > G and c.544 G > A mutations in the *SRD5A2* gene. Boxes represent exons, lines represent introns.

**Figure 4:**
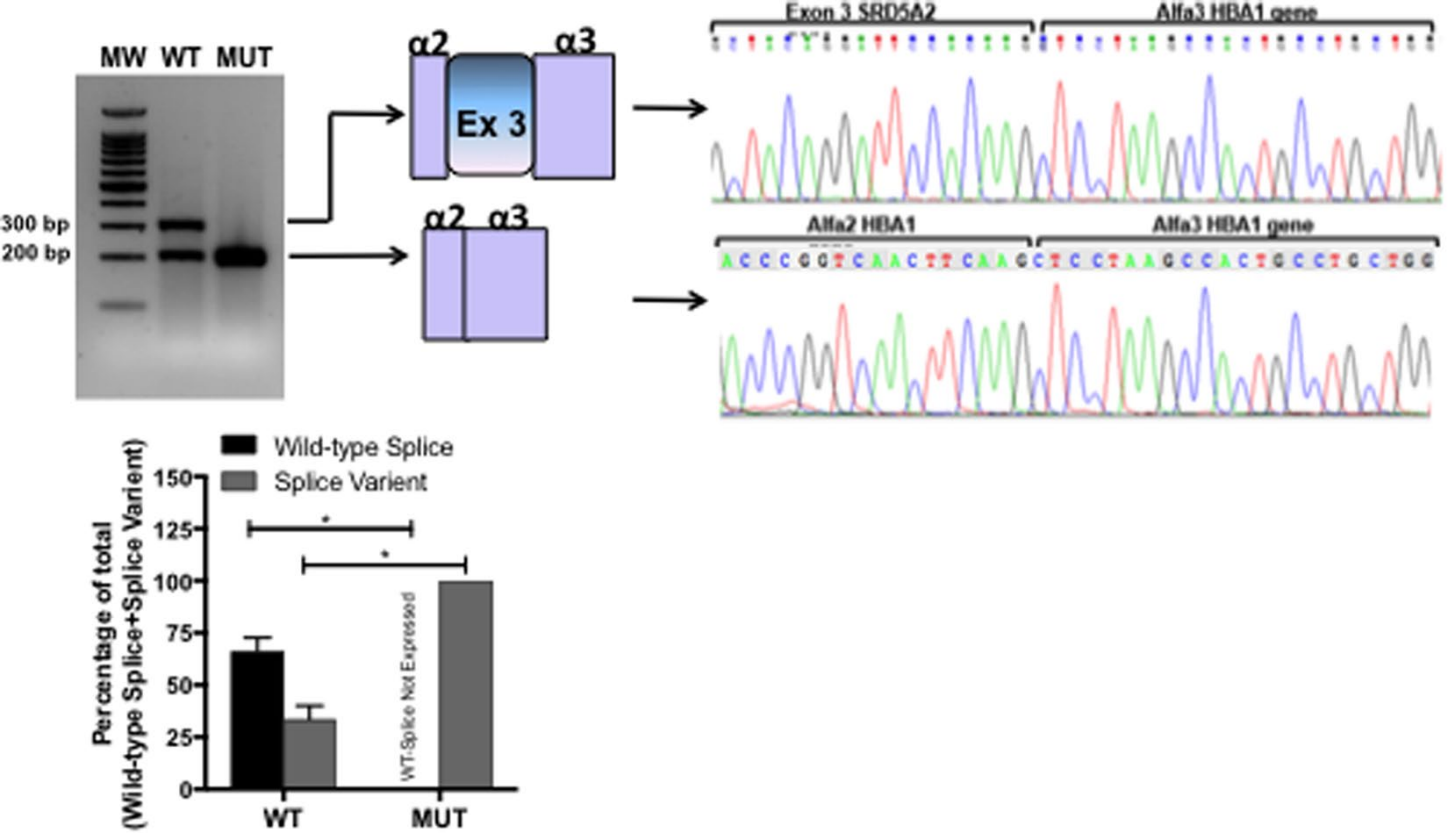
RT-PCR results from transfections of wild-type *SRD5A2* or c.544 G > A *SRD5A2* minigenes. A representative agarose gel (n = 3) showing the results of RT-PCR of wild-type (WT) and mutant (MUT) minigene splicing is shown and quantified for the accompanying graph. Schematic diagrams identifying wild-type splicing (317 bp) and exon 3 skipping (217 bp) are shown. The panel on the right shows electropherograms of cDNA splicing isoforms generated from wild-type and mutant minigenes. The histogram shows the relative percentage expression of each isoform calculated against total intensity of the bands representing each isoform. Student t test analysis shown; *p < 0.01.

The c.544 G > A *SRD5A2* mutation is located in the last nucleotide of exon 3 (5′ splice site) (Fig. 3). The DNA used for this assay was obtained from a homozygous 46,XY female patient described elsewhere^24^. Assessment of c.544 G > A minigene splicing showed that this mutation promoted skipping of exon 3 (Fig. 4), which may result in an alteration of 5α-reductase type 2 protein expression and lead to the disease phenotype.

**Figure 5:**
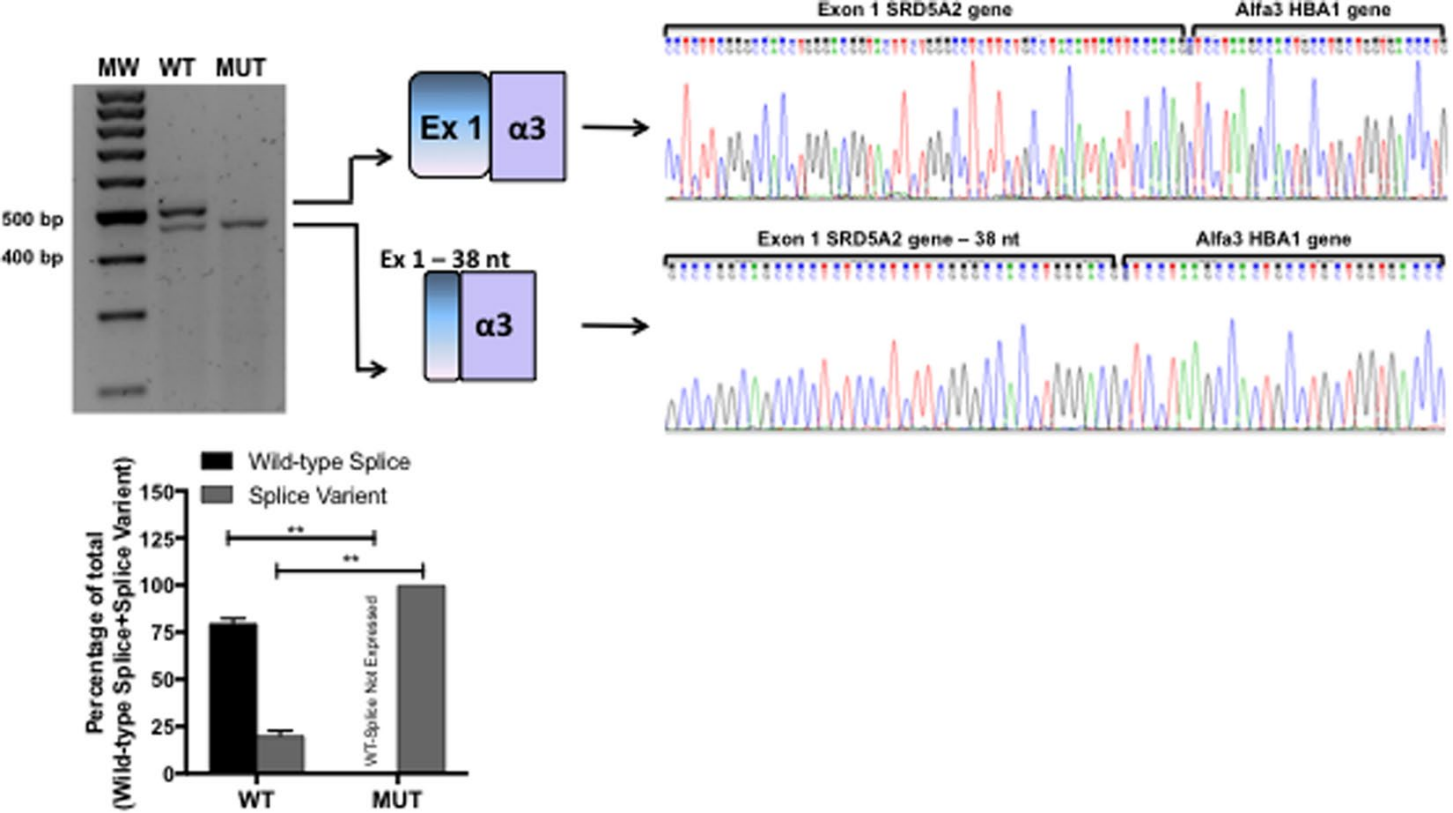
RT-PCR results from transfections of wild-type *SRD5A2* or c.278delG *SRD5A2* minigenes. A representative agarose gel (n = 3) showing the results of RT-PCR of wild-type (WT) and mutant (MUT) minigene splicing is shown and quantified for the accompanying graph. Schematic diagrams identifying wild-type splicing (479 bp) and a transcript with the deletion of 38 nucleotides (441 bp) are shown. The panel on the right shows electropherograms of cDNA splicing isoforms generated from wild-type and mutant minigenes. The histogram shows the relative percentage expression of each isoform calculated against total intensity of the bands representing each isoform. Student t test analysis shown; **P ≤ 0.001.

The c.278delG *SRD5A2* mutation is located within the 5′ splice site of exon 1 (Fig. 3). This mutation was identified in compound heterozygosis with p.Gly196Ser^21^ in a 46,XY patient with ambiguous genitalia^24^. Analysis of the amino acid sequence showed that the c.278delG mutation resulted in a frameshift that generates a premature stop codon at position 121 (p.Val81Leufs*121). Additionally, RT-PCR and sequencing results showed that c.278delG promoted the use of a cryptic 5′ splice site at c.243 resulting in a transcript with a 38-nucleotide deletion at the 3′ end of exon 1 (Fig. 5).

**Figure 6:**
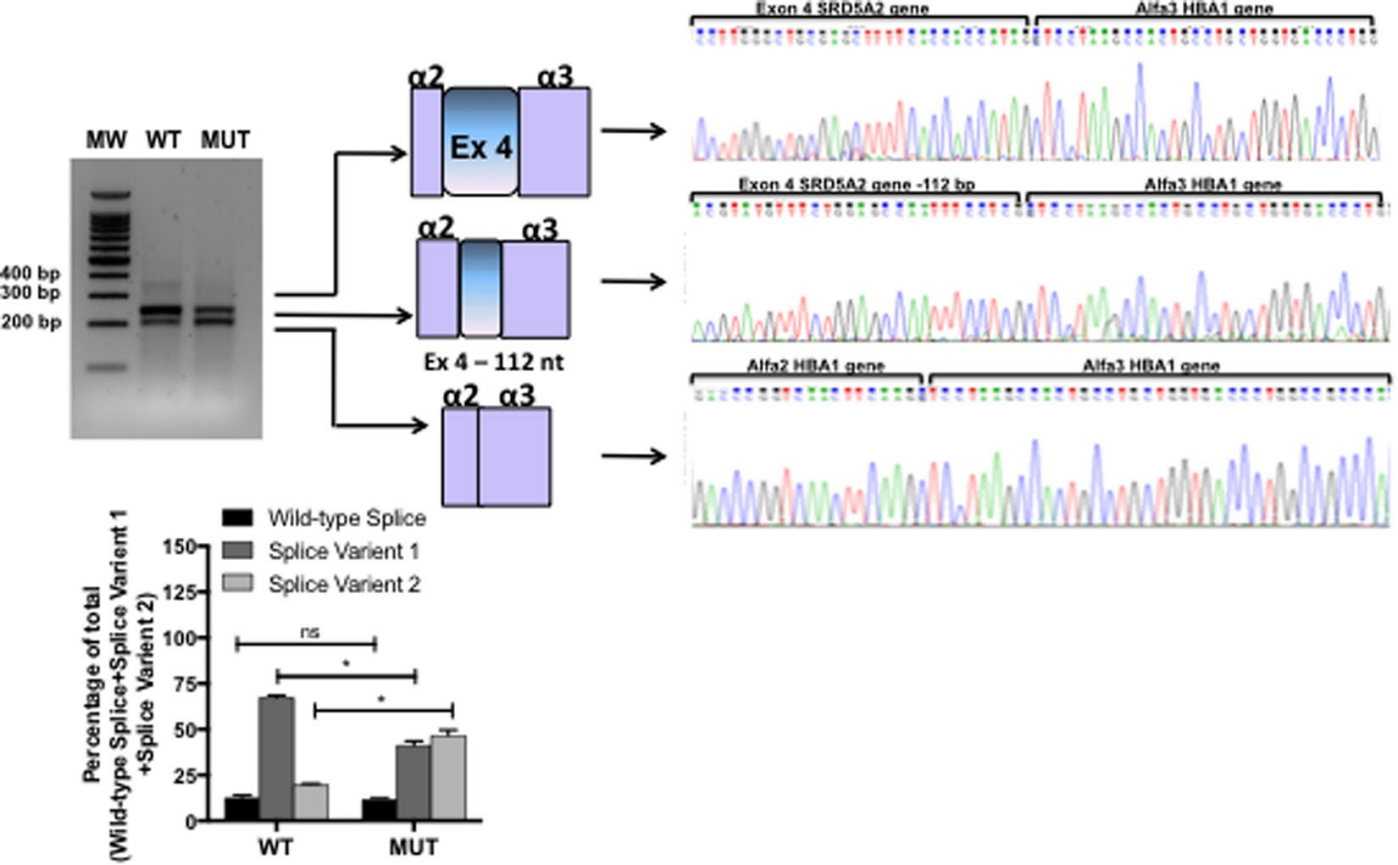
RT-PCR results from transfection of wild-type *SRD5A2* or c.548-44 T > G *SRD5A2* minigenes. A representative agarose gel (n = 3) showing the results of RT-PCR of wild-type (WT) and mutant (MUT) minigene splicing is shown and quantified for the accompanying graph. Schematic diagrams identifying the wild-type transcript (369 bp), a transcript with deletion of 112 nucleotides (257 bp), and a third transcript with exon 4 skipping are shown (217 bp). The panel on the right shows electropherograms of cDNA splicing isoforms generated from wild-type and mutant minigenes. The histogram shows the relative percentage expression of each isoform calculated against total intensity of the bands representing each isoform. Student t test analysis shown; *p < 0.01.

We evaluated the *SRD5A2* sequence variant c.548-44 T > G, located at the 3′ splice site of intron 3 (Fig. 3). This mutation was identified in a heterozygous 46,XY DSD patient that had palpable gonads in the labioscrotal folds and male genitalia. RT-PCR results showed that both WT and mutant minigenes produced three transcripts. Sequencing identified that they corresponded to: the WT transcript sequence (369 bp); a transcript with deletion of 112 nucleotides at the 3′ splice site of exon 4 (Splice Variant 1 = 257 bp); and a transcript with exon 4 skipping (Splice Variant 2 = 217 bp). The band intensity of the 3 transcripts differed between the WT and mutant c.548-44 T > G minigenes. In the WT minigene Splice Variant 1 was most prevalent (66%), whereas the mutant minigene produced both Splice Variant 1 and 2 with similar band intensity (42% and 40%, respectively) (Fig. 6) indicating that the c.548-44 T > G alteration results in an imbalance of these 3 transcripts. As it was found in simple heterozygosis, it is difficult to estimate if this contributes to the less penetrant phenotype of the patient.

## Discussion

Genetic analysis of several genes, including ataxia telangiectasia mutated (*ATM*), neurofibromin 1 (*NF1*), von Willebrand factor (VWF) and cystic fibrosis transmembrane regulator (*CFTR*), has shown that many exonic and intronic alterations, that are classified as neutral/synonymous or missense, have been subsequently found to affect the splicing process^25,26^.

It is well established that one of the earliest events in eukaryotic exon recognition by the spliceosome machinery is binding of U1 small nuclear ribonucleoprotein (snRNP) to the 5′ splice site^27,28^. U1 snRNP binds to the 5′ exon-intron junction of pre-mRNA through base pairing between the single stranded terminal sequence of the U1 RNA molecule and the loosely conserved stretch of nucleotides at the exon-intron boundary (CAG/GURAGU)^29^. Subsequent to U1 snRNP binding, ordered assembly of the four remaining snRNPs occurs to form the spliceosome, which then undergoes extensive conformational and compositional remodeling to become catalytically active^5^.

The patient bearing the c.277 + 2 T > G mutation in *HSD17B3* was compound heterozygous with the c.277 + 4 A > T and was referred to us at the age of 14 years due to lack of female secondary sexual features and signs of virilization. The patient presented with a 3-cm phallus, perineal urethral opening, and a vaginal introitus and absence of uterus indicated by ultrasound. The gonads were not palpable. The patient also had hirsutism and pubic hair stage 4 with karyotype 46,XY. Molecular findings and minigene experiments confirmed the 17β-hydroxysteroid dehydrogenase type 3 deficiency as the ethiology for 46,XY DSD in this case.

Because c.544 G > A in *SRD5A2* is located at the last nucleotide on exon 3, we wanted to identify whether its pathogenic effect could be due to aberrant splicing rather than disruption of SRD5A2 enzymatic activity caused by the missense mutation, p.Gly183Ser, as previously considered^21^. The patient, homozygous for the mutation c.544 G > A, was referred to us with 22 days of age due to genital ambiguity. The patient’s parents were first cousins and there were no other cases of genital ambiguity in the family. On physical examination the patient had a 1-cm phallus, perineal urethral opening and a vaginal introitus; there was a slight posterior labioscrotal fusion. Both gonads were palpable in the labioscrotal folds. The patient’s karyotype was 46,XY and sex assignment was female. The molecular study confirmed 5-alpha reductase type 2 gene deficiency and analysis of minigene splicing identified that the pathogenicity of this mutation may, at least partly, be the result of aberrant splicing which resulted in exon 3 skipping of *SRD5A2*.

The results so far described here support the conclusion that c.277 + 2 T > G in *HSD17B3* and c.544 G > A in *SRD5A2* potentially disrupt binding of U1snRNP to the 5′ splice site therefore inhibiting splicing initiation^29,30^. These mutations highlight the importance of a conserved 5′ splice site sequence for correct binding of U1 snRNP and spliceosome machinery assembly, and show that disruption of this process may result in human disease^30,31^.

Exonic and intronic variants may also affect splicing either by creation of new splice sites, activation of existing cryptic splice sites, or disruption/creation of other splicing regulators such as enhancers and silencers^32^. This was the case for the TP53 c.672 G > A mutation that results in activation of a cryptic 5′ splice site and the expression of an alternative splicing isoform producing a frameshift and activating a premature stop codon^33^. In this study we have described the c.278delG *SRD5A2* mutation, which sees firstly, the activation of a cryptic 5′ splice site, and secondly a frameshift that activates a premature stop codon. The patient was carrying the c.278delG mutation in compound heterozygosis with p.Gly196Ser. This patient was referred to us at the age of 5 years due to genital ambiguity. At physical examination, the patient had a 3-cm phallus, penoscrotal hypospadia, the right gonad was not palpable and the left gonad was palpable in the inguinal channel. The karyotype was 46,XY, therefore the patient was diagnosed with idiopathic 46,XY DSD. As the c.278delG minigene produced a short aberrant splicing isoform compared to WT, and also activated a premature stop codon, we can conclude that the c.278delG mutation may contribute to the observed genital ambiguity and 5alpha-reductase type 2 deficiency in this case.

Although the analysis of intronic sequence variants has been over looked for diagnostic purposes, their importance cannot be neglected as they may also affect the splicing process^3^. This study highlights the relevance of splicing analysis to verify the pathogenicity of such variants, especially those that are rare. Intronic splicing variants are often located within 50 base pairs of splice sites, but they have also been reported deep within introns where they can be more than 100 base pairs away from exon–intron boundaries^34^. For example, the deletion of 4 base pairs (GTAA) in intron 20 of *ATM*, ~3 kb from exon 20 and 0.8 kb from exon 21, leads to the activation of a pseudo exon, producing an aberrant mRNA and consequently the disease phenotype^35^. In addition, the *NF1* deep intronic single nucleotide change, c.31–279 A > G, which results in creation of a 3′ splice site, illustrate how such variants can activate cryptic 5′ splice sites and partial inclusion of a pseudoexon^36^. Mutations that inactivate or activate alternatively used splice sites could force the expression of one alternative splicing pattern over another, thus potentially favoring the expression of protein isoforms which could be incompatible with a certain cell type, developmental stage, or result in disease^37^. This was probably the case for the variant c.548–44 T > G, a very rare variant (0.2%, unpublished data) described here in a heterozygous 46,XY DSD patient. The patient was referred to us at the age of 4 years due to unilateral cryptorchidism and the older brother also had cryptorchidism. The patient presented a 4-cm phallus and typical male genitalia; the left gonad was palpable in the inguinal channel and the right gonad in the labioscrotal fold. His karyotype was 46,XY. Because of the heterozygosis of this mutation screening of other genes that could be potentially involved in the phenotype of the patient was performed. Sequencing of exons and intron/exon boundaries of *AR*, *HSD17B3*, and NR5A1 genes did not identify any significant sequence variants. Therefore we concluded that the c.548-44 T > G mutation may cause the phenotype by mediating an imbalance of isoform expression which leads directly to genital ambiguity.

Research is still unraveling many of the basic mechanisms that underlie the pre-mRNA splicing process. Clinical cases that identify mutations affecting splicing are useful for highlighting these mechanisms and how they can be disrupted in disease, which is important in diagnostic and therapeutic research. Clinically important mutations that affect the splicing process have been difficult to characterize using current diagnostic algorithms and techniques, particularly in *SRD5A2*, *HSD17B3* and other genes associated with disorders of sex development. Our findings have not only contributed to the phenotypic understanding of these patients but have also highlighted that analysis of sequence variants, not classically affecting protein function, should be assessed for their effect on splicing function to confirm pathogenicity. If RNA is readily available this analysis can be undertaken by RT-PCR, but if not available, as in this study, further confirmation can be achieved by minigene splicing assays. Analysis of splicing mutations is important for both clinical diagnosis and management, and for developing our understanding of underlying pathogenic splicing mechanisms, which will in-turn expand our knowledge of endocrine regulation in sexual differentiation.

## Materials and Methods

### Minigene construction

Patient or control DNA was used for PCR amplification with oligonucleotides carrying a non-complementary tail for specific restriction enzyme digestion. PCR products were sub-cloned into the pGEM vector (Promega) following manufacturer’s instructions. Sub-cloned PCR fragments and a pCDNA3(+) vector (Invitrogen) containing exons and introns 1–3 of the human alpha globin (HBA1) gene were digested using BamHI, EcoRI and KpnI before ligation^38^. The sequence of all minigenes were checked by sequencing.

### Cell Culture

Human Embryonic Kidney cell lines (HEK-293), were grown in DMEM medium supplemented with 1% streptomycin and 10% fetal bovine serum, and incubated in 5% CO2 at 37 °C.

### Transfection

Minigenes were transfected into HEK-293 cell lines using FuGENE HD (Roche). A mix of 100 μl of DMEM serum-free medium containing 1 ug of vector DNA and 3 μl of FuGENE reagent was incubated for 15 minutes at room temperature before being added to 6 cm-well cell cultures (60% confluent) in the presence of 10% fetal bovine serum.

### RNA Extraction and RT-PCR

RNA was extracted from cells 48 hours after transfection using an RNeasy® Plus Mini Kit (Qiagen), following manufacturer’s instructions. cDNA sysntheis was performed on 1 μg total RNA using random primers and Promgea M-MLV Reverse Transcriptase (Promega). PCR was performed on resulting cDNA using specific primers to the α-globin gene: forward primer 5′ GGACCCGGTCAACTTCAA 3′; reverse primer 5′ AGCTCTAGCATTTAGGTGACACTAT 3′, with the exception of the c.278delG mutation (*SRD5A2* gene) where the forward primer used was: 5′TAATACGACTCACTATAGGG 3′. PCR products were analyzed by electrophoresis on 1.5% agarose gels and band intensity quantified using IMAGEJ. To account for differences in product length, band intensities of each splicing isoform were normalized to the length of the wild-type splice for each minigene. The relative band intensity of each isoform was then calculated as a percentage of the total expression of all isoforms following normalization. Statistical analysis (student’s t test) was performed using GraphPad Prism software.

### cDNA Sequencing

Individual PCR fragments were purified using the GeneJET Gel Extraction Kit (Thermo Scientific). Confirmatory sequencing of RT-PCR products was performed with primers used in the PCR reaction. Chromas Lite 2.0 (Technelysium Pty) and CLC Sequence Viewer v.6.8.1 (Qiagen) were used to align and analyse cDNA and amino acid sequences against reference sequences available through the Ensembl database (SRD5A2:ENSG00000277893; HSD17B3: ENSG00000130948^39^.

### In silico analysis

Splicing regulatory sequences in *SRD5A2* and *HSD17B3* genes were predicted using the computational tools SpliceAid, and Human Splicing Finder version 2.4.1^40,41^.
